# Expression and Characterization of a Thermostable Carrageenase From an Antarctic *Polaribacter* sp. NJDZ03 Strain

**DOI:** 10.3389/fmicb.2021.631039

**Published:** 2021-03-12

**Authors:** Yuanyuan Gui, Xiaoqian Gu, Liping Fu, Qian Zhang, Peiyu Zhang, Jiang Li

**Affiliations:** ^1^College of Environmental Science and Engineering Qingdao University, Qingdao, China; ^2^Marine Bioresource and Environment Research Center, First Institute of Oceanography, Ministry of Natural Resources, Qingdao, China; ^3^Key Laboratory of Ecological Environment Science and Technology, First Institute of Oceanography, Ministry of Natural Resources, Qingdao, China; ^4^CAS and Shandong Province Key Laboratory of Experimental Marine Biology, Institute of Oceanology, Chinese Academy of Sciences, Qingdao, China

**Keywords:** carrageenase, gene expression, enzymatic characterization, enzymatic hydrolysis, antioxidant capacity

## Abstract

The complete genome of *Polaribacter* sp. NJDZ03, which was isolated from the surface of Antarctic macroalgae, was analyzed by next-generation sequencing, and a putative carrageenase gene *Car3206* was obtained. *Car3206* was cloned and expressed in *Escherichia coli* BL21(DE3). After purification by Ni-NTA chromatography, the recombinant Car3206 protein was characterized and the antioxidant activity of the degraded product was investigated. The results showed that the recombinant plasmid pet-30a-*car3206* was highly efficiently expressed in *E. coli* BL21(DE3). The purified recombinant Car3206 showed a single band on sodium dodecyl sulfate-polyacrylamide gel electrophoresis, with an apparent molecular weight of 45 kDa. The optimum temperature of the recombinant Car3206 was 55°C, and it maintain 60–94% of its initial activity for 4–12 h at 55°C. It also kept almost 70% of the initial activity at 30°C, and more than 40% of the initial activity at 10°C. These results show that recombinant Car3206 had good low temperature resistance and thermal stability properties. The optimum pH of recombinant Car3206 was 7.0. Car3206 was activated by Na^+^, K^+^, and Ca^2+^, but was significantly inhibited by Cu^2+^ and Cr^2+^. Thin-layer chromatographic analysis indicated that Car3206 degraded carrageenan generating disaccharides as the only products. The antioxidant capacity of the degraded disaccharides *in vitro* was investigated and the results showed that different concentrations of the disaccharides had similar scavenging effects as vitamin C on ${\text{O}}_{2^{•}}^{-}$, •OH, and DPPH•. To our knowledge, this is the first report about details of the biochemical characteristics of a carrageenase isolated from an Antarctic *Polaribacter* strain. The unique characteristics of Car3206, including its low temperature resistance, thermal stability, and product unity, suggest that this enzyme may be an interesting candidate for industrial processes.

## Introduction

Carrageenans are high molecular weight sulfated polysaccharides that are important components of the cell walls of red seaweeds (*Rhodophyta*) (Hjerde et al., 2017). They are composed of alternating 3-linked β-D-galactopyranose and 4-linked α-D-galactopyranose or 4-linked 3,6-anhydro-α-D-galactopyranose disaccharide repeat units (Boulho et al., 2017). Carrageenans are linear sulfated polysaccharides that have been classified into κ-, ι-, and λ-carrageenan families according to the location and number of sulfate substitutions. Among them, κ-carrageenans are the most common in industrial production.

The molecular weight of seaweed polysaccharides is an important factor that affects their biological function (Jiao et al., 2011). Therefore, compared with carrageenans, carrageenan oligosaccharides have lower molecular weights, stronger tissue penetration abilities, and more extensive application fields (Sun et al., 2015). Numerous studies have shown that carrageenan oligosaccharides have many biological roles, including antitumor (Calvo et al., 2019), antiviral (Kalitnik et al., 2013), antioxidant (Sun et al., 2010), and immunomodulation activities (Huang et al., 2020). Carrageenan degradation methods include chemical methods, such as acid hydrolysis, that usually do not keep native constituents intact (Hjerde et al., 2017); physical methods that degrade carrageenans mainly by ultrasonic, microwave, or radiation, which do not change the composition and structure of oligosaccharides, but the reaction conditions are difficult to control (Abad et al., 2009); and biological enzymatic methods with carrageenases that degrade carrageenans, which can lead to more efficient production of single stereoisomers, fewer side reactions, and a lower environmental burden (Zhao et al., 2018), and is an ideal way to obtain carrageenan oligosaccharides. However, the carrageenase-producing bacteria isolated from nature have the disadvantages of low enzyme production and poor enzyme stability (Kang et al., 2014; Barbeyron et al., 2019). Microbial enzymes that hydrolyze carrageenan are of considerable interest because enzymatic degraded products of carrageenan are still in their infancy compared with other algal polymers such as agar and alginate (Michel et al., 2001a, b, 2006; Chauhan and Saxena, 2016). Therefore, obtaining highly efficient strains, especially of bacteria isolated for special environments, which degrade carrageenans and produce highly activity carrageenases is of great value. A large number of cold-adapted marine bacteria that degrade polysaccharides have been found in extreme environments, such as the Antarctic and deep seas (Jain and Krishnan, 2017), so the characterization of microorganisms from these environments may contribute to the discovery of novel polysaccharide-degrading enzymes for industrial applications.

Studies have shown that carrageenan oligosaccharides have strong antioxidant properties *in vitro* (Yuan et al., 2006). The term antioxidant generally refers to a compound that can scavenge reactive oxygen species (ROS). There are two types of ROS, oxygen-containing free radicals, which are associated with oxygen metabolism, and peroxides, which easily form free radicals (Cadenas and Davies, 2000). Oxygen-containing free radicals include superoxide (${\text{O}}_{2^{•}}^{-}$), hydroxyl (•OH), and hydroperoxyl (HO_2_) radicals, and peroxides include hydrogen peroxide (H_2_O_2_) and lipid peroxides (ROOH). Among the ROS, •OH, and ${\text{O}}_{2^{•}}^{-}$ have the highest toxicity and cause tissue lipid peroxidation, protein depolymerization, nucleic acid fracture, and polysaccharide depolymerization, all of which can harm organisms. Therefore, the scavenging rates of •OH and ${\text{O}}_{2^{•}}^{-}$ are important indicators that reflect the antioxidant effects of antioxidants. Synthetic antioxidants are commonly used in industry, but because of toxicological safety concerns their use is restricted (Qi et al., 2005). Carrageenan oligosaccharides are expected to be developed for use in the food processing and pharmaceutical industries as natural antioxidants.

In this study, we expressed and characterized a κ-carrageenase, Car3206, that was obtained from *Polaribacter* sp. NJDZ03, which was isolated from the surface of Antarctic macroalgae. After recombinant expression, the recombinant Car3206 was purified, and its enzymatic properties were characterized. The antioxidant activity of carrageenan oligosaccharides produced by Car3206 was investigated by measuring their ability to scavenge the superoxide, hydroxyl, and DPPH (1,1-diphenyl-2-picrylhydrazyl) radicals. The results will provide a good foundation for the future research and development of marine biological resources and industrial high-value production and utilization.

## Materials and Methods

### Bacterial Strains, Plasmids, and Culture Conditions

A strain producing carrageenan lyase, *Polaribacter* sp. NJDZ03, was isolated from the surface of Antarctic macroalgae and was screened by detecting halo on carrageenan plates with Lugol’s solution (Potassium iodide and Iodine) as chromogenic agent (Supplementary Figure 1). The strain *Polaribacter* sp. NJDZ03 preserved in the Key Laboratory of Marine Bioactive Substances, First Institute of Oceanography, Ministry of Natural Resources (Qingdao, China). This carrageenase-producing strain was grown at 15°C in a medium containing 0.5% tryptone, 0.1% yeast extract, and seawater. *Escherichia coli* BL21(DE3) (Tiangen Bio, Co., Ltd., Beijing, China) and *E. coli* DH5α (Tiangen) containing the pET-30a(His•Tag) plasmid (Takara Bio Inc., Beijing, China) were grown at 37°C in Luria–Bertani (LB) medium containing 10% tryptone, 5% yeast extract, and 10% NaCl or LB agar supplemented with kanamycin (50 mg/mL).

### Cloning and Analysis of the Recombinant Car3206 Nucleotide and Amino Acid Sequences

Genomic DNA was extracted from *Polaribacter* sp. NJDZ03 using a Genomic DNA Extraction Kit (Takara, Shiga, Japan) and used as the template of PCR amplification. The forward and reverse primers were 5′-CGCCATATGATTAATAAATATAA AAGCGCA-3′ (underlined bases are the added *Nde*I site) and 5′-CCCAAGCTTCTGTATTGGTTATTGCTGCC-3′ (underlined bases are the added *Hind*III site). The PCR system (50 μL) was as follows: Master Mix (Tiangen) 25 μL, forward and reverse primers 1 μL each, template 1 μL, and ddH_2_O 22 μL. The PCR conditions were: 94°C for 2 min, and 35 cycles of 94°C for 30 s, 56°C for 30 s, and 72°C for 1.5 min, with a final extension step held at 72°C for 10 min. The PCR products were purified using a DNA purification kit (Takara, Shiga, Japan), then ligated into pET-30a(His•Tag), which was digested previously with *Nde*I (Takara) and *Hind*III (Takara). The constructed expression plasmid was transformed into *E. coli* DH5α cells. The transformed cells were screened using LB agar supplemented with kanamycin (50 mg/mL) at 37°C for 12 h, Then, the positive colonies were picked and put into the LB medium with kanamycin (50 mg/mL) at 37°C for 12 h. The PCR products were sequenced by the Shanghai Sangon Company.

The nucleotide and amino acid sequences were analyzed using GeneMark software^1^. BlastP and BlastN^2^ searches were performed to identify the sequences and Motif Search^3^ was used for motif analyses. The DNAMAN software package^4^ was used for multiple sequence alignment.

### Expression and Purification of the Recombinant Car3206 Protein

The *Car3206* gene was ligated into a pET-30a expression vector and transformed into *E. coli* BL21(DE3) cells. The cells harboring the pET30a–*Car3206* recombinant plasmid were grown in LB medium containing kanamycin (50 μg/mL) on a rotary shaker (150 rpm) at 37°C. When the OD_600_ reached 0.6, isopropyl-β-D-thiogalactopyranoside (IPTG) (0.5 mM) was added to the LB medium. Cultivation was continued for 16 h at 16°C and 120 rpm. Then, the cells were harvested by centrifugation (4°C, 7,500 × *g*, 20 min), resuspended in phosphate buffer (0.2 M Na_2_HPO_4_-NaH_2_PO_4_, pH 7.0), and disrupted using an ultrasonic cell disruptor (Biosafer, China). Cell debris was removed and the supernatant was obtained by centrifugation (4°C, 10,000 × *g*, 10 min). The target protein was purified using a His Bind Purification Kit (GE Healthcare, Co., Ltd., United States) and washed with binding buffer (20 mmol/L sodium phosphate, 0.5 mol/L NaCl, 20 mmol/L imidazole, pH 7.4). Then, the recombinant Car3206 was eluted using elution buffer (20 mM sodium phosphate, 500 mM NaCl, pH 7.4) with a linear gradient to 250 mM imidazole (Riera et al., 2003). The purity of the eluted recombinant Car3206 protein was analyzed by 12% sodium dodecyl sulfate-polyacrylamide gel electrophoresis (SDS-PAGE).

### Assay of Car3206 Enzyme Activity

The enzymatic hydrolysis reaction system was as follows: 200 μL carrageenase was incubated with 1,800 μL phosphate buffer (0.2 M Na_2_HPO_4_-NaH_2_PO_4_, pH 7.0) containing 0.1% (w/v) carrageenan at 55°C for 45 min. The reducing oligosaccharide products in the reaction mixture were assayed using the 3,5-dinitrosalicylic acid method (Miller, 1959). One unit of enzyme (U) was defined as the amount of carrageenase needed to liberate 1 μM reducing sugar (measured as D-galactose) from carrageenan per min. All assays were performed in triplicate.

### Characterization of the Recombinant Car3206 Protein

The optimum temperature for recombinant Car3206 activity was determined under standard assay conditions by varying the incubation temperature from 10 to 90°C. As the control, the highest enzyme activity was defined as 100%. The thermal stability of recombinant Car3206 was determined by incubating the enzyme solution at 45, 55, or 65°C for different times (1, 2, 3, 4, 5, 12, and 24 h) then measuring the residual enzyme activity under the standard assay conditions. As the control, the highest enzyme activity was defined as 100%.

The effect of pH on the recombinant Car3206 activity was assayed by incubating with 0.1% (w/v) carrageenan dissolved in different buffer systems, namely Na_2_HPO_4_-citric acid (pH 4.0–7.0), 0.05 M Tris–HCl (pH 7.1–8.9), and 0.05 M glycine-NaOH (pH 9.0–10.6) under standard assay conditions. As the control, the highest enzyme activity was defined as 100%.

The effects of various metal ions and a reagent on the activity of the recombinant Car3206 were determined by monitoring enzymatic activity in the presence of 5 mM of metal ions (Mn^2+^, Sr^2+^, Fe^2+^, Fe^3+^, Mg^2+^, Ca^2+^, Cr^2+^, Cu^2+^, Na^+^, and K^+^) and EDTA. Each test was conducted under standard assay conditions. As the control, the carrageenase activity without the metal ions or reagent was set as 100%.

### Analysis and Preparation of Enzymatic Hydrolysis Products

Enzymatic hydrolysis products of carrageenan were determined by thin layer chromatography. We incubated 2 mL purified enzyme with 8 mL phosphate buffer (0.2 M Na_2_HPO_4_-NaH_2_PO_4_, pH 7.0) containing 0.1% (w/v) carrageenan at 55°C for 0.5, 1, and 6 h. The solutions were boiled for 10 min, then centrifuged (4°C, 10,000 × *g*, 10 min). The products at the different reaction times were added to Silica gel 60 F_254_ thin-layer chromatography plates (Merck, Germany) with the developing system (n-butanol:ethanol:water = 2:1:1, v/v/v). The resultant oligosaccharide spots were visualized by spraying with 10% (v/v) H_2_SO_4_ in ethanol and heating at 80°C for 15 min.

In addition, the 6-h hydrolysis product was collected, filtered, and concentrated using an Amicon Ultra-15 centrifugal filter unit (Millipore, Germany). The filtrate was evaporated, lyophilized, and collected as the oligosaccharide products for the antioxidant assay.

### Antioxidant Activity of Enzymatic Hydrolysate

#### Scavenging Assay of Superoxide Radical (${\text{O}}_{2^{•}}^{-}$)

The scavenging assay of ${\text{O}}_{2^{•}}^{-}$ was based on a modified phenazine-methosulfate (PMS)-NADH method (Siddhuraju and Becker, 2007). Briefly, 468 μM NADH, 60 μM PMS, and 150 μM NBS were prepared with phosphate buffer (0.1 M Na_2_HPO_4_-NaH_2_PO_4_, pH 7.4). Then, 1 mL NADH, 1 mL PMS, 0.5 mL carrageenan oligosaccharide samples at different concentrations, and 1 mL nitroblue tetrazolium (NBT) were added into the test tube in turn and mixed. The mixed solution was incubated at room temperature for 6 min, then the absorbance of each mixed solution was measured at 560 nm. The measurement was repeated three times and the results were averaged. The blank control group with distilled water instead of carrageenan oligosaccharide also was measured under the same conditions, and vitamin C (Vc) was used as the positive control. The concentration of Vc was 0.5 mg/mL and the concentrations of carrageenan oligosaccharides were 0.5, 1, 2, 4, and 8 mg/mL.

The scavenging capacity of a carrageenan oligosaccharide sample for the superoxide anion radical (${\text{O}}_{2^{•}}^{-}$) was calculated as:

$$\mathrm{Scavenging}\mathrm{effect}=\frac{{\text{A}}_{0}-{\text{A}}_{1}}{{\text{A}}_{0}}\times 100\%$$

where A_0_ is the absorbance measured in the reaction solution of the blank control group and A_1_ is the absorbance measured in the reaction solution of each experimental group.

#### Scavenging Assay of Hydroxyl Radical (•OH)

FeSO_4_ can react with H_2_O_2_ to form hydroxyl radicals (•OH). The reaction equation is:

$$\text{Fe}{}^{2+}+\text{H}{}_{2}\text{O}{}_{2}\;\to \text{ Fe}{}^{3+}+\text{OH}{}^{-}+•\text{OH}$$

The hydroxyl radical scavenging capacity was determined by the method of Jin et al. (2005) with modifications. Briefly, 1.5 mL of 5.0 Mm 1,10-phenanthroline solution (95% ethanol as solvent) was mixed with 2.0 mL of phosphate buffer (0.2 M Na_2_HPO_4_-NaH_2_PO_4_, pH 7.4), then 1.0 mL of 7.5 mM FeSO_4_ solution was added. The solution was evenly mixed and 1.0 mL of 0.1% (w/v) H_2_O_2_ was added to start the reaction. Finally, redistilled water was added to a total volume if 10 mL, and this reaction system was used as the control group. For the experimental group, 1.0 mL Vc or carrageenan oligosaccharide at different concentrations was added, then 0.1% (w/v) H_2_O_2_ was added to start the reaction. Vc was used as a positive control. The mixture without scavengers (Vc or carrageenan oligosaccharides) and H_2_O_2_ was used as the blank control. The absorbance of each reaction solution was measured at 536 nm after incubation at 37°C for 1 h. The Vc concentration was 0.5 mg/mL, and the carrageenan oligosaccharide concentrations were 0.5, 1, 2, 4, and 8 mg/mL.

The scavenging capacity of a carrageenan oligosaccharide sample for hydroxyl radicals (•OH) was calculated as:

$$\mathrm{Scavenging}\mathrm{effect}=\frac{{\text{A}}_{\text{c}}-{\text{A}}_{\text{a}}}{{\text{A}}_{\text{b}}-{\text{A}}_{\text{a}}}\times 100\%$$

where A_a_ is the absorbance measured in the reaction solution of the control group, A_b_ is the absorbance measured in the blank control, and A_c_ is the absorbance measured in the reaction solution of each experimental group.

#### Scavenging Assay of DPPH Radical

The DPPH radical scavenging ability was assessed by the method of Sun and Ho (2005). Briefly, 2 mL of 0.15 mM DPPH radical (95% ethanol as solvent) was mixed with 2 mL carrageenan oligosaccharide at different concentrations. The Vc concentration was 0.5 mg/mL and the carrageenan oligosaccharide concentrations were 0.5, 1, 2, 4, and 8 mg/mL.

The scavenging capacity of carrageenan oligosaccharides to hydroxyl radicals (•OH) was calculated as:

$$\mathrm{Scavenging}\mathrm{effect}=\left(1-\frac{{\text{A}}_{\text{i}}-{\text{A}}_{\text{j}}}{{\text{A}}_{0}}\right)\times 100\%$$

where A_*i*_ is the absorbance of the mixed solution measured at 517 nm after reacting in the dark for 30 min, A_*j*_ is the absorbance measured after the DPPH solution in the reaction system was replaced with 95% ethanol, and A_0_. is the antioxidant (oligosaccharide sample or Vc) in the reaction system with distilled water instead.

## Results

### Sequence Analysis and Expression of *Car3206*

A carrageenase-encoding gene, *Car3206*, was obtained from the whole genome of *Polaribacter* sp. NJDZ03 isolated from the surface of Antarctic macroalgae. The open reading frame of *Car3206* consisted of 1,203 bp, which encoded a protein of 400 amino acids with a calculated molecular weight of 44.5 kDa. Sequence similarity showed that the encoded carrageenase Car3206 belonged to the glycoside hydrolase family 16 (GH16). To our knowledge, this is the first report of a carrageenase isolated from an Antarctic *Polaribacter* strain.

**FIGURE 1:**
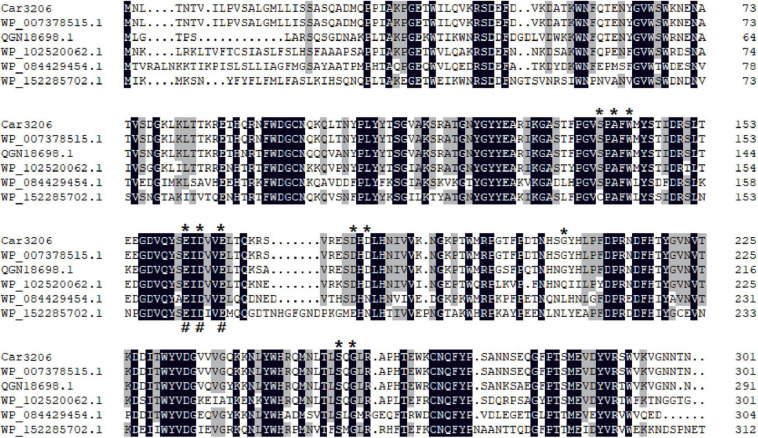
Multiple sequence alignment of Car3206 protein sequences. The predicted κ-carrageenase amino acid sequence of *Car3206* was aligned with the deduced amino acid sequences of κ-carrageenase genes from *Pseudoalteromonas* sp. (GenBank: WP_007378515.1), *Vibrio* sp. (GenBank: QGN18698.1), *Shewanella* sp. 10N.286.48.A6 (GenBank: WP_102520062.1), *Aliagarivorans marinus* (GenBank: WP_084429454.1), and *Flavicella marina* (GenBank: WP_152285702.1). *indicates active sites; #indicates catalytic sites; black columns indicate identical amino acids; and gray columns indicate amino acids with ≥50% identity.

The amino acid sequence of *Car3206* from the *Polaribacter* sp. strain shared high similarity with the sequences of other reported carrageenases. Multiple sequence alignment showed that the Car3206 sequence shared 90.75% homology with the orthologous sequence of *Pseudoalteromonas* sp. (GenBank: WP_007378515.1), 80.99% with *Vibrio* sp. (GenBank: QGN18698.1), 68.58% with *Shewanella* sp. 10N.286.48.A6 (GenBank: WP_102520062.1), 57.05% with *Aliagarivorans marinus* (GenBank: WP_084429454.1), and 56.66% with *Flavicella marina* (GenBank: WP_152285702.1). Based on the multiple alignment and NCBI’s conserved domain database, 11 residues make up the active site (Ser_139_, Ala_141_, Trp_143_, Glu_162_, Asp_164_, Glu_167_, Asp_178_, Asp_180_, Gly_206_, Ser_255_, and Gly_257_) and three residues make up the catalytic site (Glu_162_, Asp_164_, and Glu_167_) (Figure 1; Michel et al., 2001a).

**FIGURE 2:**
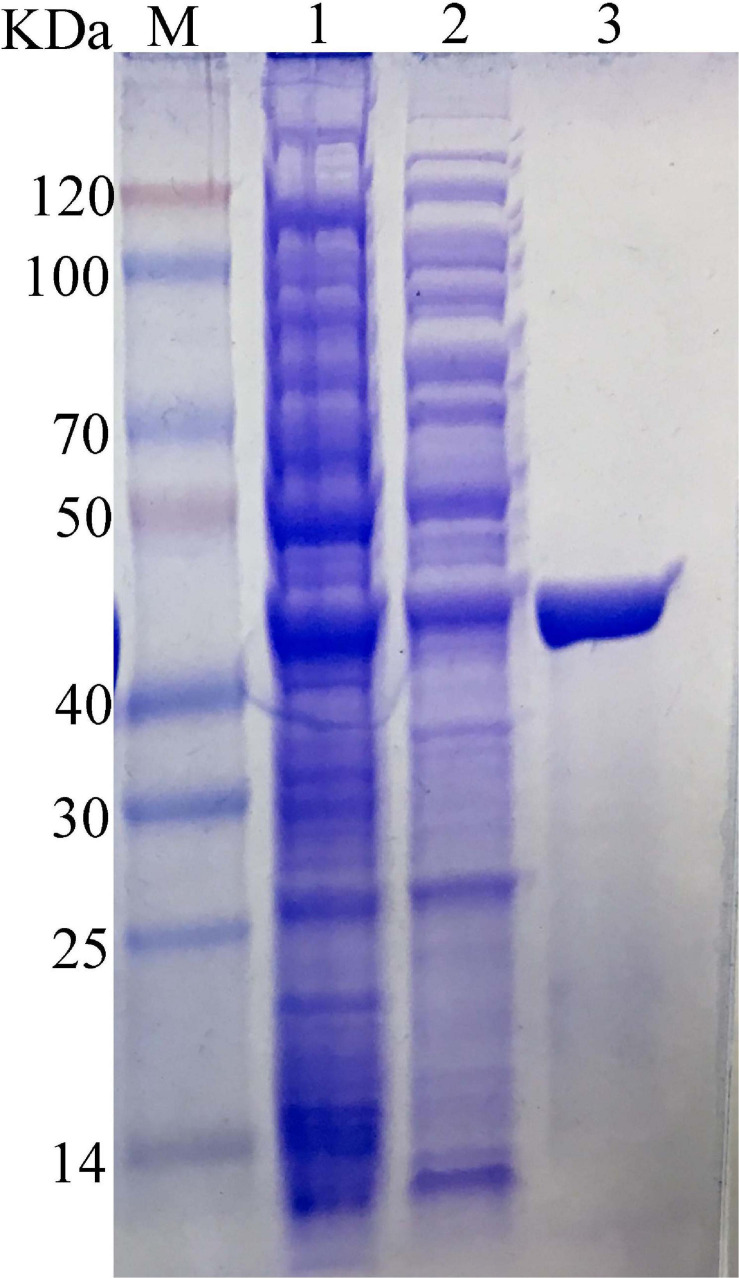
SDS-PAGE of the purified recombinant carrageenase Car3206. M, protein marker; Lane 1, recombinant *Escherichia coli* BL21(DE3) cells harboring pET30a-Car3206 after induction; Lane 2, uninduced recombinant *E. coli* BL21(DE3) cells harboring pET30a-Car3206; Lane 3, purified recombinant Car3206.

The carrageenase gene *Car3206* was expressed in *E. coli* BL21 (DE3) cells harboring pET-30a-Car3206 by IPTG induction. The crude carrageenase Car3206 was purified by Ni-NTA His Tag Kit affinity chromatography and analyzed by SDS-PAGE. A single band with an apparent molecular weight of 45 kDa was found (Figure 2), which is consistent with the calculated molecular weight.

### Characterization of the Recombinant Car3206 Protein

**FIGURE 3:**
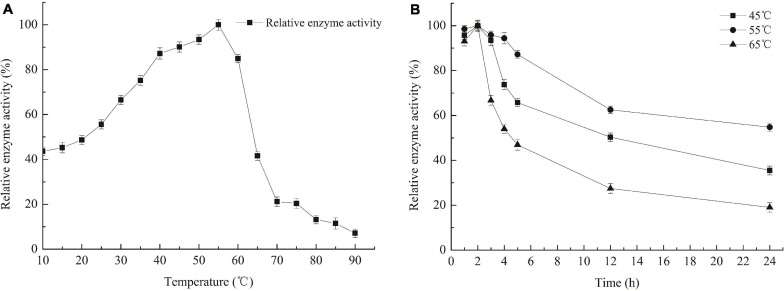
Optimal temperature **(A)** and temperature stability **(B)** of recombinant carrageenase Car3206.

**FIGURE 4:**
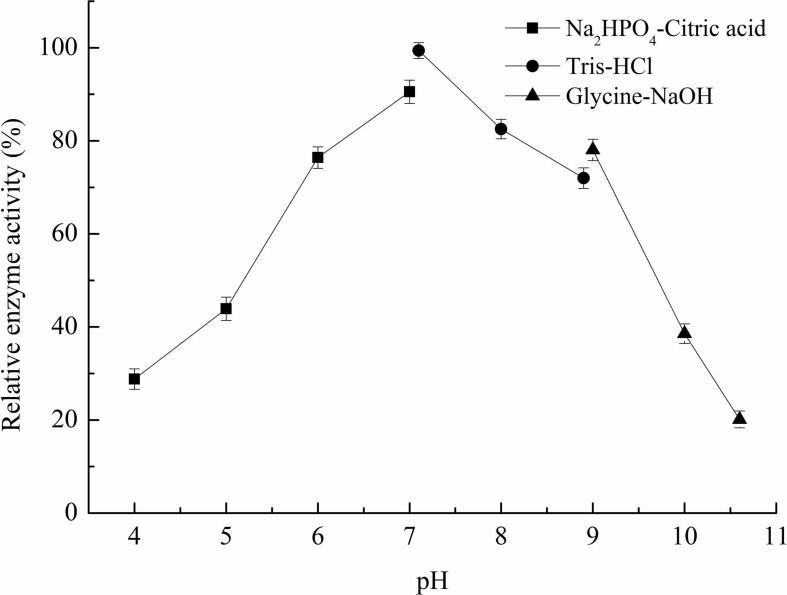
Optimal pH of recombinant carrageenase Car3206.

The optimum temperature for Car3206 enzyme activity was 55°C (Figure 3). However, 44–90% activity was retained at low temperatures (10–45°C) and >85% activity was retained at 45–60°C, but at temperatures >60°C, the enzyme activity dropped sharply (Figure 3). The thermal stability analysis showed that recombinant Car3206 retained >90% of its enzyme activity after incubation at 45°C for 3 h and 50% of its enzyme activity after incubation for 12 h (Figure 4). At the optimal temperature of 55°C, 94% of the enzyme activity was retained after incubation for 3 h and 60% was retained after incubation for 12 h, which indicated that Car3206 had good thermal stability and low temperature tolerance.

The optimum pH for Car3206 enzyme activity was 7.0. Recombinant Car3206 was stable from pH 5.0–10.0, retaining >40% of the activity in this pH range (Figure 4).

**FIGURE 5:**
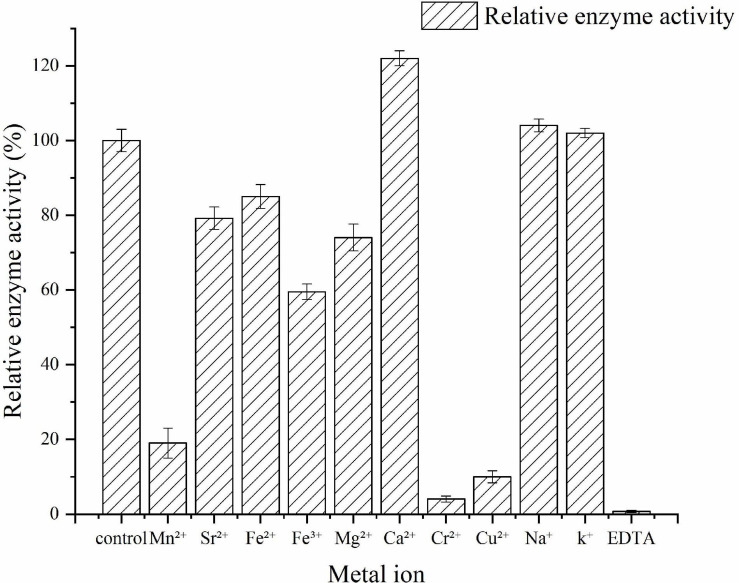
Effects of metal ions on recombinant carrageenase Car3206.

The effects of metal ions on Car3206 enzyme activity are shown in Figure 5. Ca^2+^ significantly stimulated the enzyme activity, and Na^+^ and K^+^ slightly increased its activity. Conversely, Mn^2+^, Sr^2+^, Fe^2+^, Fe^3+^, and Mg^2+^ decreased Car3206 activity, and Cr^2+^ and Cu^2+^ sharply reduced its activity by 90%. EDTA is a chelating agent that almost inactivated Car3206, implying that metal ions play crucial role in Car3206 enzyme activity.

### Hydrolysis Products of Carrageenan by Car3206 Activity

**FIGURE 6:**
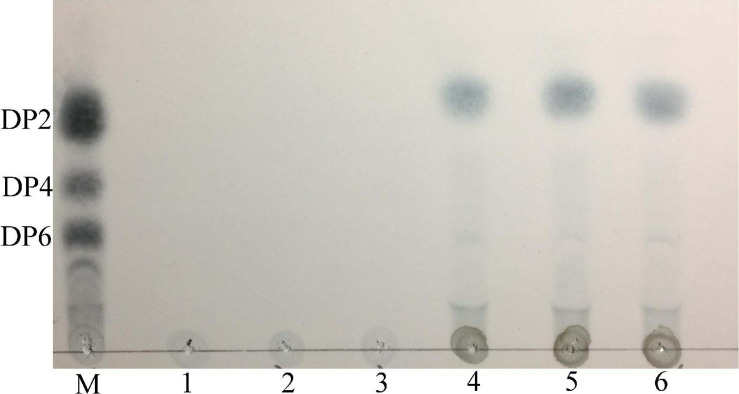
Thin layer chromatography of the hydrolysis products of purified recombinant Car3206. M, standard mixture of carrageenan, disaccharide (DP2), tetrasaccharide (DP4), and hexasaccharide (DP6); Lane 1, recombinant Car3206; Lane 2, the substrate (0.1% carrageenan); Lane 3, products of carrageenan with inactivated Car3206 for 0.5 h; Lane 4, products of carrageenan with Car3206 for 0.5 h; Lane 5, products of carrageenan with Car3206 for 1 h; Lane 6, products of carrageenan with Car3206 for 6 h.

The thin layer chromatography result showed that Car3206 effectively degraded carrageenan (Figure 6). The only products were disaccharides and no intermediate products were detected. This result demonstrated that recombinant Car3206 hydrolyzed carrageenan to disaccharides as the end products through an exo-type mode of action.

### Antioxidant Activity of the Hydrolysis Products

**FIGURE 7:**
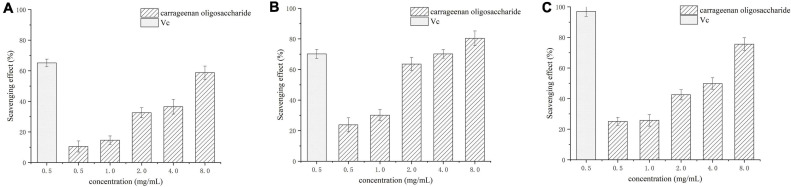
Scavenging effect of κ-carrageenan oligosaccharide on superoxide radicals **(A)**, hydroxyl radicals **(B)**, and DPPH radicals **(C)**. Vc, vitamin C.

Assays of scavenging superoxide, hydroxyl, and DPPH radicals were used to assess the antioxidant activity. The superoxide, hydroxyl, and DPPH radicals scavenging activities of carrageenan oligosaccharides at concentrations of 0.5–8 mg/mL are shown in Figure 7. The results showed that all tested concentrations of the carrageenan oligosaccharide had scavenging effects on ${\text{O}}_{2^{•}}^{-}$, •OH, and DPPH•, and the scavenging rate of enzymatic hydrolysis to the three kinds of radical increased with increasing carrageenan oligosaccharide concentrations. All these results indicated that the enzymatic hydrolysis products had similar antioxidant activity with vitamin C.

## Discussion

The development of marine bio-active substances has led to a focus on the biological activity of oligosaccharides. Oligosaccharides degraded from carrageenan are increasingly being used in medical, food, and industrial applications (Zhu et al., 2020). Carrageenan oligosaccharides have been reported many times for their antioxidant activity and potential medicinal value (Sun et al., 2015; Calvo et al., 2019). These oligosaccharides are expected to be used as natural antioxidants to replace synthetic antioxidants that are potential safety hazards. Therefore, it is urgent to find novel microorganisms that produce carrageenases with high activity and high yield originated from special environment. Macroalgae are an important source of biomass in the marine environments of Antarctica, meanwhile, they are also a valuable source of novel and exploitable carbohydrate-active enzymes (Loque et al., 2010; Hettle et al., 2019). Antarctic macroalgae host complex and diverse microbiology communities. Among them, agarolytic and carrageenolytic strains have important ecological roles as agents to decompose the algal biomass in the marine environment of Antarctica. In this study, the carrageenase gene *Car3206* was obtained from the whole genome of *Polaribacter* sp. NJDZ03, which was isolated from the surface of Antarctic macroalgae. To our knowledge this is the first reported carrageenases isolated from an Antarctic *Polaribacter* strain. This result will deepen our knowledge of biodiversity of polysaccharide degrading bacteria, and provide a new idea for future research on the function and role of polysaccharide degrading bacteria in the marine carbon metabolism system of the Southern Ocean.

The extreme environment of cold and dry has created Antarctic microorganisms with special characteristics for them to thrive. For example, they produce cold-active or cold-adaptive enzymes that have been used in mechanism studies and are potential enzymes for commercial development (Bull et al., 2000). The genus *Polaribacter* is unique to the polar region. It means that *Polaribacter* sp. strain could use carrageenan of macroalgae as carbon source and decompose it into oligosaccharides by secreting carrageenan degrading enzyme, which is an important way for algae to return carbon fixed by photosynthesis to the atmosphere. Namely, *Polaribacter* sp. plays a role in the carbon cycle of the Southern Ocean. This result deepened our understand that these bacteria play an important role in the decomposition of algae, and on nutrient cycling dynamics in Antarctic marine ecosystems (Quartino et al., 2015). The special living environment in polar regions makes Car3206 have higher cold tolerance than other carrageenan degrading bacteria. Car3206 could maintain 44–67% of its maximal activity between 10 and 30°C (Figure 3A), indicating the cold-adapted feature of this enzyme. This feature allows enzymatic reactions to be carried out at lower temperatures and has the advantage for special applications, such as recovery of nucleic acid from low concentration agarose.

The cold-adapted GH16 κ-carrageenase Cgk16A has been reported to have a maximum activity of 52% at 15°C, but the activity quickly vanished at 30 and 40°C (Shen et al., 2018). Car3206 retains 44–90% activity at 10–45°C, unlike other carrageenases, indicating that Car3206 has both a high optimum temperature and good low temperature tolerance. Reaction temperature and thermostability are very important biochemical characteristic for industrial applications. Our results showed that the optimum temperature of the recombinant carrageenase Car3206 was 55°C. Most of the characterized carrageenases have maximum activity around 40–45°C; for example, the optimum temperature of κ-carrageenase S942 from *Pseudoalteromonas* sp. is 37°C (Lin et al., 2018), for the κ-carrageenase PpCgkCD from *Pseudoalteromonas porphvrae* LL1 it is 40°C (Zhao et al., 2018), and for κ-carrageenase PLJ30 from *Pseudoalteromonas carrageenovora* ASY5 is 45°C (Xiao et al., 2018; Table 1). However, the κ-carrageenase CgkX from *Pseudoalteromonas* sp. QY203 (Xu et al., 2015) and *P. carrageenovora* ASY5 (Li et al., 2016) have optimum temperatures similar to that of Car3206 (Table 1).

**TABLE 1:** Comparison of Car3206 with carrageenases from other bacterial strains.

Enzyme	Source	Temperature	pH	Cation activators	Cation inhibitors	Products	References
Car3206	*Polaribacter* sp. NJDZ03 popolarba	55°C	7.0	Ca^2+^, Na^+^, and K^+^	Mn^2+^, Sr^2+^, Fe^2+^, Fe^3+^, Mg^2+^, Cr^2+^, and Cu^2+^	Disaccharide	This study
S942	*Pseudoalteromonas* sp.	37°C	7.0	Ca^2+^, Na^+^, and Mg^2+^	Cu^2+^, K^+^, Zn^2+^, and Mn^2+^	–	Lin et al., 2018
PpCgkCD	*Pseudoalteromonas porphyrae* LL1	40°C	8.0	Ca^2+^, K^+^, and Mg^2+^	Mn^2+^, Fe^2+^, Fe^3+^, Zn^2+^, Cu^2+^, and Hg^2+^	Neo-κ-carrabiose and neo-κ-carratetraose	Zhao et al., 2018
PLJ30	*Pseudoalteromonas carrageenovora* ASY5	45°C	6.5	Ag^+^ and Cd^2+^	Sn^2+^, Ba^2+^, Ca^2+^, Al^3+^, Mg^2+^, Sr^2+^, Mn^2+^, Zn^2+^, Fe^2+^, and Fe^3+^	Disaccharide and tetrasaccharide	Xiao et al., 2018
CgkX	*Pseudoalteromonas* sp. QY203	55°C	7.0	Na^+^	Zn^2+^, Cu^2+^, Ca^2+^, Mg^2+^, Al^3+^, NH${}_{4}^{+}$, Fe^3+^, Ca^2+^, and K^+^	Disaccharide and tetrasaccharide	Xu et al., 2015
κ-carrageenase	*Pseudoalteromonas carrageenovora* ASY5	55°C	9.0	Na^+^, K^+^, Sn^2+^, and Fe^3+^	Cd^2+^, Ba^2+^, Mg^2+^, Fe^2+^, and Ca^2+^	–	Li et al., 2016

At high concentrations, carrageenan is highly viscous at room temperature and is an inhibitor of carrageenase degradation. The most commonly used method for obtaining low-viscosity carrageen is to increase the solution temperature. However, most reported carrageenan degrading enzymes are not stable above 40°C. Therefore, to prepare oligo-carrageenan on a commercial scale, it is necessary to select superior producer bacterial strains to improve the yield and thermostability of carrageenases (Li et al., 2019). The thermal stability results showed that Car3206 retained 96% activity after incubation at 55°C for 3 h, and 60% activity after incubation at 55°C for 12 h. The optimum temperature of κ-carrageenase PpCgkCD (Zhao et al., 2018) from *P. porphvrae* LL1 is 40°C, above 45°C the enzyme activity decreased sharply; when incubated at 40°C for 1 h it retained about 80% of its maximum activity, but enzyme activity was completely lost after incubation at 50°C for 1 h. The recombinant κ-carrageenase PLJ30 (Xiao et al., 2018) was stable after incubation at 45°C for 30 min and retained 80% of its maximum activity, when incubated at 50°C for 30 min it retained approximately 50% of its maximum activity, but lost approximately 80% of its activity after incubation at 45°C for 2 h. The κ-carrageenase CgkX (Xu et al., 2015) exhibited the outstanding stability at 0–40°C, where over 90% of the total activity was remained after the 1 h thermal treatment. However, the CgkX was almost inactivated after incubation at 50°C for 1 h. And the κ-carrageenase from *P. carrageenovora* ASY5 (Li et al., 2016) retained 95% of its activity after incubation at 40°C for 1 h, and retained 60% of its maximum activity after incubation at 45°C for 1 h. Compared with other carrageenases, Car3206 has good thermal stability and high optimal temperature, which are the characteristics needed for industrial applications; making Car3206 a good candidate for industrial use (Table 2).

**TABLE 2:** Comparison of optimum temperature and thermal stability between Car3206 and other κ-carrageenases.

Enzyme	Source	Optimal temperature	Incubation conditions	relative activity	References
Car3206	*Polaribacter* sp. NJDZ03	55°C	55°C for 3 h 55°C for 12 h	96% 60%	This study
PpCgkCD	*Pseudoalteromonas porphyrae* LL1	40°C	40°C for 1 h 50°C for 1 h	80% 0%	Zhao et al., 2018
PLJ30	*Pseudoalteromonas carrageenovora* ASY5	45°C	45°C for 30 min 50°C for 30 min	80% 50%	Xiao et al., 2018
CgkX	*Pseudoalteromonas* sp. QY203	55°C	0–40°C for 1 h 50°C for 1 h	¿90% 0%	Xu et al., 2015
κ-carrageenase	*Pseudoalteromonas carrageenovora* ASY5	55°C	40°C for 1 h 45°C for 1 h	95% 60%	Li et al., 2016

Metal ions had different effects on the carrageenase activity of recombinant Car3206 (Table 1). Car3206 activity improved significantly in the presence of Ca^2+^, which is consistent with the results of a previous report (Liu et al., 2013). Ca^2+^ has significant effects on the activity of most known carrageenase, probably by forming metal–carbonyl coordination bonds with amino acids in the active centers of enzymes, thereby stabilizing the three-dimensional structure of the protein. Low concentrations of most metals, including Na^+^ and K^+^, are known to increase carrageenase activity (Li et al., 2013, 2014), especially high concentrations of Na^+^ (up to 500 mmol/L) (Ma et al., 2013). Conversely, Cu^2+^ has a potential inhibitory effect on most carrageenases possibly because it competes with Ca^2+^ for the binding sites in the active centers of the enzymes; however, the exact catalytic mechanism needs further study.

In the study of the immune defense mechanism of carrageenan oligosaccharides, Guo et al. (2017) found that κ-carrageenan disaccharide can directly enter cells and affect cellular immune regulation because of its small molecular weight and less sulfate group. Therefore, the preparation of high quality carrageenan disaccharides has a great application prospect in the field of medicine. Oligo-carrageenan produced by the action of microbial enzymes can be more advantageous than produced by acid hydrolysis because enzymes are highly specific to their substrates and they generate oligo-derivatives are uniform in molecular weights (Yao et al., 2014). But Microbial enzymes which hydrolyze carrageenan is still in its infancy compared with that of other hydrocolloids such as agar and alginate (Chauhan and Saxena, 2016). The recombinant carrageenase Car3206 effectively degraded carrageenan into disaccharides. Both the recombinant carrageenase PLJ30 from *P. carrageenovora* ASY5 and CgkX from *Pseudoalteromonas* sp. QY203 degrade carrageenan into disaccharide and tetrasaccharide, the κ-carrageenase PpCgkCD from *P. porphvrae* LL1 degrades carrageenan into neo-κ-carrabiose and neo-κ-carratetraose (Table 1). Most carrageenase degradation products are mixtures, which makes it difficult to purify each type of oligosaccharide, whereas Car3206 produces only disaccharides, which can significantly reduce the cost of product purification in industrial production.

Wang et al. (2011) found that the antioxidant activity of carrageenan oligosaccharides with low sulfate content was better than that of those with high sulfate content, and low molecular weight oligosaccharides were shown to have higher antioxidant properties (Zhou et al., 2014), implying that the neocarrabiose from κ-carrageenan degradation may effectively promote antioxidant activity in cells and eliminate the damage caused by free radicals. We investigated the antioxidant capacity of the carrageenan disaccharides produced by Car3206 *in vitro* by measuring the scavenging effects on superoxide, hydroxyl, and DPPH radicals at concentrations of 0.5–8 mg/mL. The results showed that, at different concentrations, the disaccharides had obvious scavenging effects on ${\text{O}}_{2^{•}}^{-}$, •OH, and DPPH, and the scavenging effect tended to increase with increasing concentrations of the disaccharides. The scavenging capacity of the carrageenan disaccharides was similar to that of Vc at the same concentration, implying that this degraded product of carrageenan had high antioxidant potential.

In our study, the carrageenase gene *car3206* was successfully expressed in *E. coli* and high proportion soluble recombinant protein was obtained (Figure 2). Furthermore, recombinant Car3206 has an optimal activity at 55°C and more than 80% relative activity at 60°C which is important for applications where high concentration of carrageenan is required because at high temperature it is more soluble as well as more oligosaccharides production. Moreover, Car3206 showed good thermal stability which still maintained 60% activity after incubation at 55°C for 12 h, we know, the thermostability of the enzyme is another main factors for limiting its industrial production., In addition, the final degradation product of Car3206 is only disaccharide, which greatly simplify the separation process and reduced the production cost. In conclusion, our study also provides new ideas for the development and utilization of Antarctic microbial resources. The antioxidant capacity of carrageenan degradation products provides a path for research and industrial utilization of natural antioxidants in the future.

## Data Availability Statement

The raw data supporting the conclusions of this article will be made available by the authors, without undue reservation.

## Author Contributions

JL collected the samples, designed research, supervised the project, and analyzed data. YG performed the experiments and wrote the manuscript. XG, LF, and QZ performed the experiments and prepared figures. PZ analyzed the data. All authors contributed to the article and approved the submitted version.

## Conflict of Interest

The authors declare that the research was conducted in the absence of any commercial or financial relationships that could be construed as a potential conflict of interest.

## Footnotes

1 http://opal.biology.gatech.edu/GeneMark/

2 http://blast.ncbi.nlm.nih.gov/Blast.cgi

3 http://www.genome.jp/tools/motif/

4 http://www.lynnon.com/
