# Supplementary material for: Expression and Characterization of a Thermostable Carrageenase From an Antarctic Polaribacter sp. NJDZ03 Strain
Source: Front Microbiol. 2021 Mar 12;12:631039. doi: 10.3389/fmicb.2021.631039 (PMC7994522; doi:10.3389/fmicb.2021.631039)
Supplement: Supplementary file 1 [file Table_1.DOC]

Supplementary Figures

#
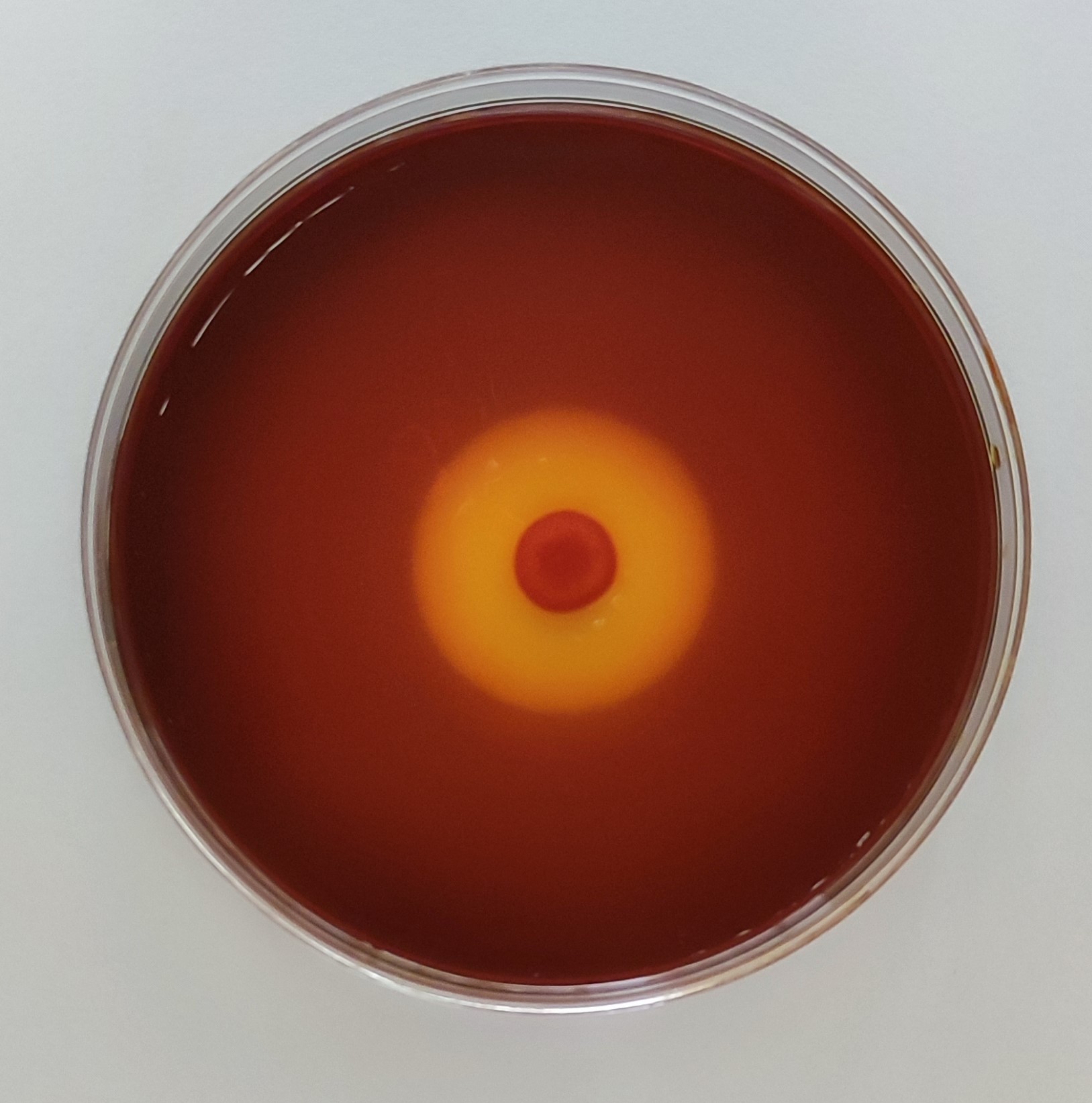


**Supplementary Figure 1.** Isolation of carrageenan degrading strains *Polaribacter* sp. NJDZ03

Lugol’s solution staining, showing the distinct zones of clearance of the carrageenan degrading strains *Polaribacter* sp. NJDZ03.
